# Neuroblastoma of the lumbosacral canal in an adult: a case report and literature review

**DOI:** 10.3389/fneur.2023.1195664

**Published:** 2023-08-03

**Authors:** Qingyu Jiang, Haihao Gao, Gan Gao, Yang Li, Haofeng Cheng, Guoliang Shi, Aijia Shang

**Affiliations:** ^1^Chinese PLA Medical School, Beijing, China; ^2^Department of Critical Care Medicine, Chinese PAP Beijing Corps Hospital, Beijing, China; ^3^Medical School, Nankai University, Tianjin, China; ^4^Department of Neurosurgery, Chinese PLA General Hospital, Beijing, China

**Keywords:** neuroblastoma, lumbosacral canal, surgery, tumor, neurological deficits

## Abstract

Neuroblastoma (NB) is a leading cause of death in children. It usually occurs in the adrenal gland and rarely in the spinal canal. Here, we report the case of a 48-year-old male patient with abnormal thickening of the cauda equina nerve as revealed by lumbosacral magnetic resonance imaging. The patient’s main clinical manifestations were numbness and pain in both lower limbs. The patient underwent surgical treatment; however, intraoperatively, an unclear border was observed between the cauda equina nerve and the tumor; therefore, the tumor was not forcibly excised. The postoperative pathological results were reported as NB. The disease known as NB, which is extremely rare. We believe that a pathological biopsy is extremely vital for diagnosing NB, and aggressive post-operative radio-chemotherapy could potentially prolong the patient’s survival time.

## 1. Introduction

Neuroblastoma (NB) is the most common extracranial solid tumor in children, with a higher prevalence in males than in females and a higher mortality rate among younger children (1, 2). NB comprises approximately 9% of childhood cancers and is rare in adults (3–7). Compared to children, most adults are diagnosed when the cancer has already metastasized (8). NBs can occur throughout the sympathetic nervous system, most commonly in the adrenal gland (47%), abdominal/retroperitoneal region (24%), and sympathetic ganglia (9). Nevertheless, it is rare in other regions of the sympathetic nervous system (10), particularly NBs originating in the adult adrenal medulla, which are extremely rare (11, 12). Here, we report a rare case of an adult patient with spinal NB.

## 2. Case description

The patient was a 48-year-old male (height, 160 cm; weight, 49 kg) who was not engaged in heavy physical work and had a history of smoking for 10 years, with an average of one pack daily. He had no history of alcohol consumption. No obvious causes of eyelid droop and blurred vision were found 1 year before admission to our hospital, and the neostigmine test was positive. Titin and RyR antibody statuses were positive, and his local hospital diagnosed him with myasthenia gravis. Brombistigmine 60 mg was administered three times daily. The patient reported some relief in his effectiveness; however, 3 months before admission to our hospital, his lower limb weakness had worsened and was accompanied by numbness; accordingly, he was referred to our hospital for treatment. Physical examination showed that the patient had clear consciousness, fluent speech, and normal memory and orientation; furthermore, bilateral gastrocnemius tenderness was present, muscle strength was grade 5 for both upper limbs, grade 5 for the proximal left lower limb, and grade 4 for the right lower limb, and grades 4 and 3 for the back and plantar flexion, respectively. The tendon reflexes of both upper limbs were normal; the knee-tendon reflex of both lower limbs presented hyperreflexia. Ankle reflex decreased, the lateral side of both lower legs and the superficial sensation of the dorsum of the foot decreased, deep tenderness beside the spinous process of the L4-5 intervertebral space was accompanied by lower limb radial pain, and the deep sensation below both iliac arteries was slightly decreased.

## 3. Diagnostic assessment

**Figure 1:**
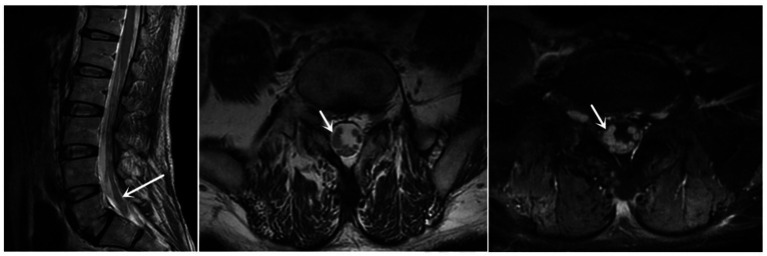
Sagittal and axial MRI revealed that the cauda equina was thickened, and enhanced MRI revealed that the cauda equina was significantly enhanced (at the white arrow). MRI, magnetic resonance imaging.

**Figure 2:**
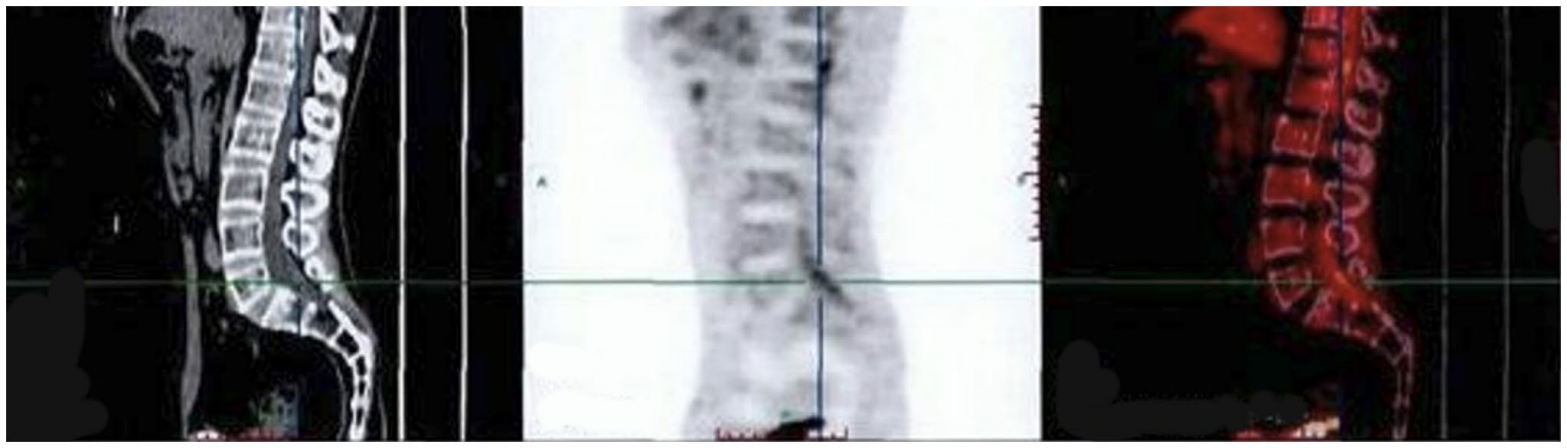
PET/CT indicating that the cauda equina in the spinal canal is thickened. SUVmax (Standardized Uptake Value): 2.7. PET/CT, positron emission tomography/computed tomography.

The finger-nose and heel–knee-shin test results were normal, and a bilateral positive Babinski sign was observed. Based on the patient’s symptoms, including lower limb weakness and numbness, we speculated that the patient may have malignancy, infections, or cauda equina syndrome (13, 14). Therefore, we suggested that he undergo a magnetic resonance imaging (MRI) of the lumbosacral region to observe if any lesions exist within the spinal canal. Lumbosacral MRI showed abnormal thickening of the cauda equina nerve in the spinal canal at the L5-S2 level (Figure 1), and the shape and location of the masses were relatively rare. Due to the rapid progression of the patient’s condition, malignancy in the lumbosacral region was comparable, although the possibility of central nervous system infection cannot be ruled out. Therefore, it was recommended that the patient undergo positron emission tomography/computed tomography (PET/CT) and have his cerebrospinal fluid collected. PET/CT showed that the cauda equina in the spinal canal was thickened, and multiple linear radioactive concentrations were present (Figure 2). Cerebrospinal fluid examination revealed a monocyte reaction and heterologous cells were positive. To rule out the possibility of tumor metastasis in the intracranial and other parts of the spinal canal, we recommended that the patient undergo an MRI of the cranium, cervical, and thoracic spine; the results revealed no obvious abnormalities. Subsequently, the patient was transferred to the neurosurgery department for tumor removal.

**Figure 3:**
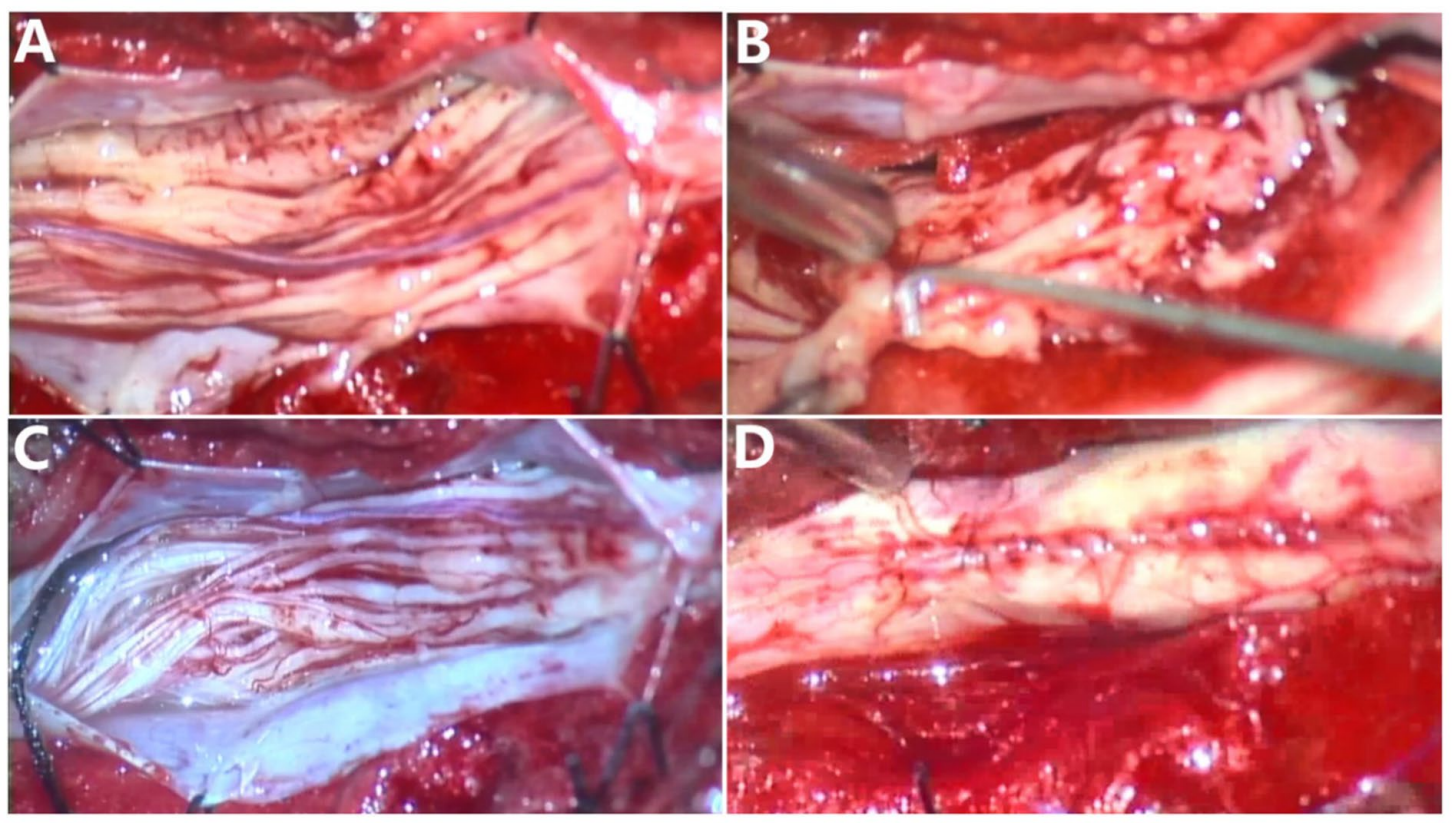
**(A)** The cauda equina nerve is surrounded by tumors, yellow-white, soft, and rich in blood supply. **(B)** Blunt stripping of the tumor and cauda equina nerve. **(C)** Sufficient hemostasis after biopsy. **(D)** Suture of the dura mater.

The patient was placed in a prone position, and an L5-S2 skin incision was made on the spinous process; the subcutaneous and fascia were cut layer-by-layer, the muscles were separated and pulled on both sides, and the spinous process and vertebral lamina were completely ground down through piezosurgery. After the dura mater and arachnoid membrane were excised, the tumors were mainly located at the S1 and S2 levels and originated in the cauda equina. Approximately two-thirds of the cauda equina nerve in the sacrum showed tumor growth with different thicknesses, yellow-white color, soft texture, and rich blood supply. The larger tumor on the right side was removed; however, no obvious boundary was observed between the tumor and nerve root, and complete removal was not achieved. Finally, the tumor tissue (approximately 2.5 × 0.3 × 0.3 cm in size) was removed and sent for pathological examination. Other tumors were small, and the border with the nerve root was unclear; therefore, they were not treated forcibly. After careful hemostasis, they were returned to the spinal processes of the vertebral lamina (Figure 3).

**Figure 4:**
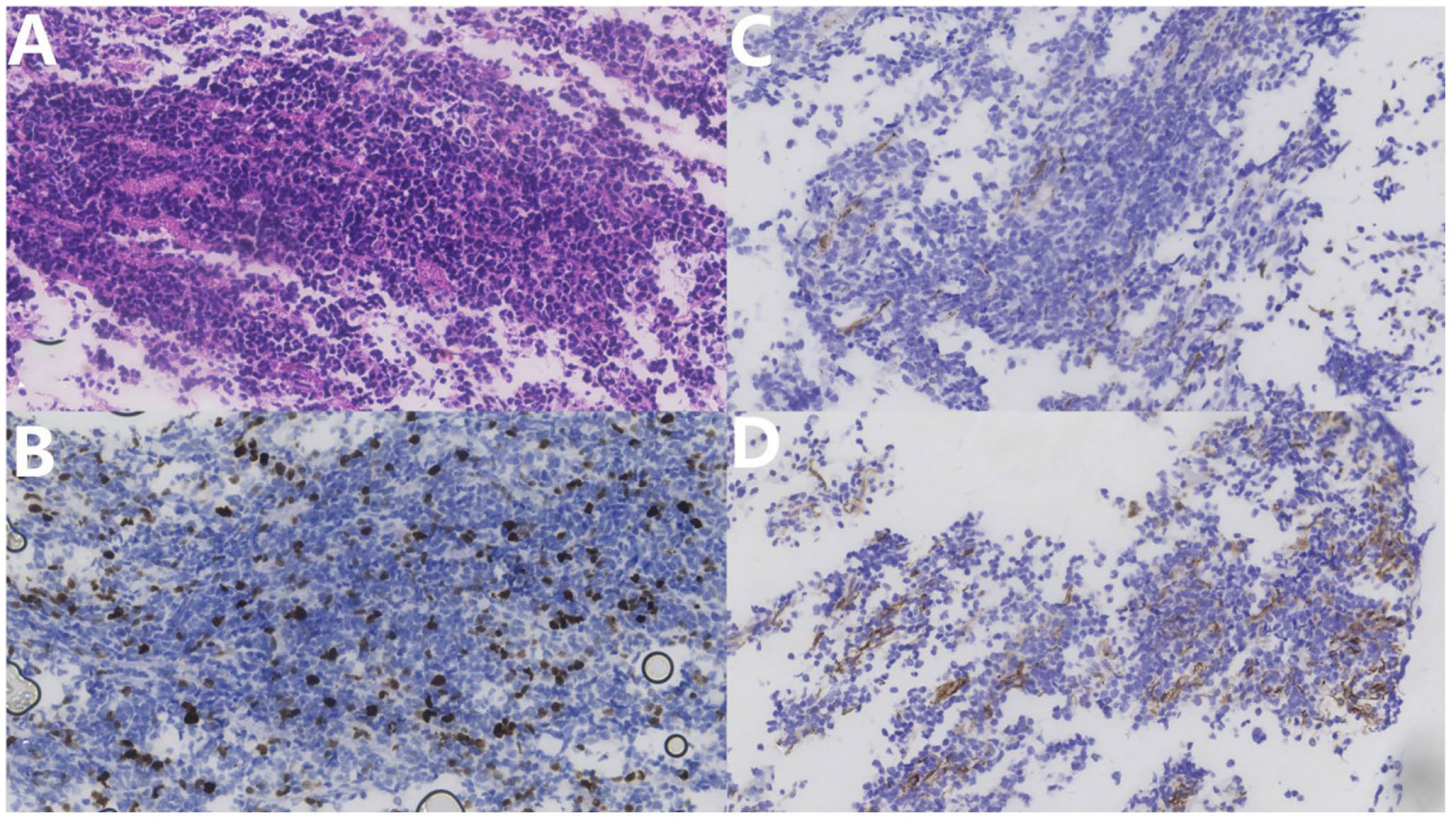
**(A)** Hematoxylin–eosin stain (×400) shows tumor cell sheet distribution, moderate atypia, eosinophilic cytoplasm, oval cells, nuclear staining, some visible nucleoli, and no clear necrosis. **(B)** Ki67 immunohistochemical staining (×400) shows approximately 30% positive rate. **(C)** NF immunohistochemical staining (×400). **(D)** NFD Nestin immunohistochemical staining (×400) shows a partial positive rate.

Pathological examination showed the sheet distribution of cells, moderate atypia, eosinophilic cytoplasm, oval cells, nuclear staining, and some visible nucleoli; however, no clear necrosis was found (Figure 4). Immunohistochemical investigations suggested that the biopsy tissue cells expressed CK (–), Ki-67 (+30%), TTF-1 (–), Syn (+), CgA (–), CD3 (T cell +), CD20(scattered B cell +), CD56 (+), CD99 (partial +), NeuN (–), MAP-2 (+), GFAP (–), Nestin (–), NF (–), β-catenin (+), NSE (+), C-myc (–), p53 (–), and Olig-2 (individual cells +). Based on the histopathological pattern, the tumor was diagnosed as NB, World Health Organization grade IV. The multidisciplinary consultation suggested that whole central radiotherapy combined with chemotherapy (cyclophosphamide + topotecan + doxorubicin + vincristine + cisplatin + etoposide) should be performed. At discharge, the patient’s general condition was acceptable. The patient complained that the symptoms of weakness in both lower limbs were slightly worse than those at the time of admission and accompanied by pain in both lower limbs. The incision on the back of the waist healed well postoperatively. Physical examination revealed that the muscle strength of both upper limbs was grade 5. The proximal and distal muscle strength of the left and right lower limbs were grade 4. Bilateral Pap signs were negative. Late in the treatment, the patient could not tolerate the radiotherapy and chemotherapy due to severe bone marrow suppression and refused further immunotherapy attempts, such as anti-gd2. After 8 months of follow-up, he died of a severe pulmonary infection. His total survival time was 22 months.

## 4. Discussion

Here, we summarize the most relevant origins, genetic and chromosomal alterations, clinical manifestations, and management of NB.

### 4.1. Origins and genetic alterations

#### 4.1.1. Origins

A recently detailed review suggested that adrenal chromaffin cells and sympathetic neurons are reliable cell sources for NB (15). Moreover, neural crest cells are a source of NB cells (16). In vertebrates, the neural crest is a transient cell group that produces various other cell groups under the control of a complex gene regulatory network (17). Because NBs mainly occur in the adrenal gland, sympathetic adrenal progenitor cells are considered the source of NBs. Furthermore, because primary NB occurs in the paravertebral sympathetic ganglia in addition to the adrenal gland, Schwann cell precursors are considered one of the origins of NB (15).

#### 4.1.2. Genetic alterations

The DNA index (DI) is used to express DNA content. Typically, the DI of diploid cells is 1.0; when the DI >1.0, hyperdiploidy is considered present. Additionally, the DNA content of tumor cells is related to the pathogenesis of NB; in children with NB aged < 18 months, “patients with a DI > 1.0 had a lower tumor stage and better prognosis than those with a DI of 1.0” (18).

#### 4.1.3. Chromosomal alterations

Pugh et al. sequenced genomic DNA samples from 240 high-risk NBs and found that chromosome changes were the most common (19). Segmental chromosomal aberrations associated with poor prognosis included *MYCN* amplification, 17q gain, 11q loss, and 1p36 loss (20). *MYCN* and *let-7* miRNA families have recently been shown to be closely related (21); *let-7* miRNAs play essential roles in inhibiting tumor growth and development. Power et al. believed that the expansion of *MYCN* competitively inhibits *let-7* miRNA, weakening its tumor inhibition function (22). This hypothesis may explain why *MYCN* amplification is associated with advanced disease, rapid tumor progression, and poor prognosis (23). Regarding 17q gain, six of the 14 samples showed an unbalanced gain of one to three additional copies of 17q (24). Moreover, *MYCN* overexpression may promote an increase in the 17q chromosome. Therefore, an increase in 17q is associated with stronger tumor invasiveness, which is usually used as a genetic predictor of patient prognosis (25, 26). Approximately 30% of NB cases have 11q deletion. *MYCN* amplification and 11q deletion are the most relevant genetic markers of high-risk tumors (27). Moreover, 11q deletion, *MYCN* amplification, and tumor ploidy are the only NB markers used to determine treatment plans (28). Finally, 1p encodes one or more NB tumor suppressor genes; the most commonly missing region is located in 1p36 (29, 30).

### 4.2. Clinical manifestation

Posterior mediastinal NBs usually form a mass outside the dura mater. Approximately 10–15% of the tumor tissue invades the spinal canal, infiltrating the intervertebral foramen with or without spinal cord compression (31, 32). According to the degree of spinal cord compression, 7%–10% of patients may have back pain, motor disorders, sphincter dysfunction, and sensory disorders (33, 34). More than half of these patients experience permanent sequelae, which are more common in young patients (35, 36).

### 4.3. Management

Early decompression of the spinal cord through neurosurgery can prevent irreversible neurological changes (37, 38). Most patients who underwent thoracic surgery and neurosurgery combined approach had complete subsidence postoperatively; therefore, this method may be safe and effective for treating mediastinal and intraspinal NB (39). However, laminectomy should be considered carefully because some patients present spinal deformities postoperatively (40, 41). Although spinal braces have become popular postoperatively, their impact remains unclear. For instance, they may delay the occurrence of spinal deformities rather than reduce them (42). Therefore, chemotherapy is the first choice, particularly in young children, for reducing the long-term sequelae of laminectomy or spinal radiation. Emergency neurosurgical decompression is only performed in the case of neuro-progression during chemotherapy (31, 43). However, in 30% of patients who received chemotherapy, neurological function did not improve and even showed signs of deterioration (43, 44). Optimal treatment for patients with epidural compression remains controversial. In some studies (37), patients receiving chemotherapy did not require further neurosurgical intervention or radiotherapy to relieve secondary compression. However, the efficacy of various treatment methods has been evaluated, and the combination of surgery and radiotherapy was associated with the greatest functional improvement (45). Long before chemotherapy was proven effective, decompression neurosurgery and radiotherapy were used to treat symptomatic spinal cord injury (46). However, 57% of children receiving radiotherapy had radiation-induced spinal deformities (47). Additionally, more severe deformities are associated with an increased radiation dose, longer follow-up time, and younger age. Therefore, radiotherapy is rarely used currently (6, 35). Furthermore, dexamethasone in spinal cord compression has been demonstrated to reduce edema, inhibit inflammatory reactions, stabilize vascular membranes, and delay the occurrence of nerve function defects (48). Some studies have shown that moderate-to-high doses of dexamethasone can be recommended for patients with significant neurological dysfunction (49). Currently, no consensus exists on the best treatment strategy for intraspinal NB (50, 51).

Krol and Horten reported only a case of NB with primary cauda equina nerve in 1980 (52). Because of the rapid progression of NB and its variable location, the atypical clinical symptoms lead to the difficulty of initial accurate diagnosis. Similar to this case, 16% of adults are misdiagnosed during their first visit, resulting in a delay in receiving appropriate treatment (53). Failure to correctly diagnose may be due to the rare incidence of NB, rare MRI scan performance, and atypical clinical symptoms. The case we reported started with numbness and weakness in the lower limbs, which was consistent with the growth site of the tumor. The MRI scan of this case showed thickening of the cauda equina roots, an inhomogeneous slightly high signal in T1WI and T2WI, and no obvious abnormality in the pressure-fat image. The cauda equina was unevenly and moderately enhanced on the enhanced scan, and its signal was coarse on the axial scan. Although the MRI scan was typical of NB, differentials for tumors in the cauda equina included the following: schwannoma (the tumor is mostly regular round-like and has a clear boundary with the cauda equina nerve); lipoma (patients with spinal cord tethered cord syndrome have filum terminale steatosis and sacral lipoma, and MRI shows thickening of the cauda equina, but MRI lipogram conducive to exclude); lymphoma (would show homogeneous enhancement in MRI scan, and inclined to invade the surrounding vertebral body); and metastatic tumors (the MRI appearance of metastatic tumors may be similar to that of NB; however, they commonly destroy the vertebral bodies and accessories and have primary lesions). The MRI manifestations of this case were specific, and the tumor growth mode differed from that of the spinal cord NB in previous reports. Commonly, NB causes spinal cord injury mostly due to the invasion of the tumor into the spinal canal through the intervertebral foramen, resulting in compressive damage to the spinal cord (37). In this case, it originated from the cauda equina, which could be observed using MRI enhancement scanning that the involved cauda equina was strengthened as a whole without a clear boundary. Intraoperatively, we found that the boundary between the tumor tissue and cauda equina nerves was blurred.

This surgical procedure had the following advantages: intraoperatively, it was observed that many cauda equina nerves in the sacral canal adhered closely to the tumor. Considering the patient’s postoperative quality of life, no forced resection was performed; therefore, no significant damage was caused to the cauda equina nerves. The limitation is that the tumor may not have been completely excised; therefore, future recurrence is possible. A study conducted by Hung et al. reported on the tentative use of chemotherapy drugs, including carboplatin, etoposide, cyclophosphamide, and doxorubicin for neoadjuvant chemotherapy in adult male patients with NB; reexamination showed that the patients’ tumors shrank by approximately 17% (54). Unfortunately, this patient did not receive chemotherapy. Therefore, we highly recommend that patients undergo chemotherapy and regular follow-up to monitor tumor growth after biopsies.

## 5. Conclusion

Adult NB originates from the adrenal medulla, which is extremely rare. MR imaging and clinical symptoms may not show obvious features; therefore, a pathological biopsy may be necessary for diagnosis. Due to the overall poor prognosis of this disease, and the lack of clear boundaries between tumor tissue and the adrenal medulla, surgical resection can be challenging. However, aggressive chemotherapy followed by maintenance therapy may be beneficial in prolonging patient survival.

## Data availability statement

The original contributions presented in the study are included in the article/supplementary material, further inquiries can be directed to the corresponding author.

## Ethics statement

The studies involving human participants were reviewed and approved by Ethics Committee of Chinese PLA General Hospital and don’t contain identifiable human images. The patients/participants provided their written informed consent to participate in this study. Written informed consent was obtained from the participant/patient(s) for the publication of this case report.

## Author contributions

QJ and GG wrote the main manuscript text. YL, GS, and HC prepared Figures 1–3. HG prepared Figure 4 and provided interpretations of the pathological results and made revisions to the article. All authors contributed to the article and approved the submitted version.

## Funding

This study was funded by Capital’s Funds for The National Key Research and Development Program of China (grant number 2022YFC2703304) and Health Improvement and Research (grant number CFH2022-2-5022).

## Conflict of interest

The authors declare that the research was conducted in the absence of any commercial or financial relationships that could be construed as a potential conflict of interest.

The reviewer LD declared a shared affiliation with the authors to the handling editor at the time of review.

## Publisher’s note

All claims expressed in this article are solely those of the authors and do not necessarily represent those of their affiliated organizations, or those of the publisher, the editors and the reviewers. Any product that may be evaluated in this article, or claim that may be made by its manufacturer, is not guaranteed or endorsed by the publisher.
